# P120 Catenin Isoforms Differentially Associate with Breast Cancer Invasion and Metastasis

**DOI:** 10.3390/cancers11101459

**Published:** 2019-09-29

**Authors:** Jan-Hendrik Venhuizen, Paul N. Span, Koen van den Dries, Sebastian Sommer, Peter Friedl, Mirjam M. Zegers

**Affiliations:** 1 Department of Cell Biology, Radboud Institute for Molecular Life Sciences (RIMLS), Radboud University Medical Center, 6525 GA Nijmegen, The Netherlands; Jan-Hendrik.Venhuizen@radboudumc.nl (J.-H.V.); Koen.vandenDries@radboudumc.nl (K.v.d.D.); sebastian_sommer@icloud.com (S.S.); Peter.Friedl@radboudumc.nl (P.F.); 2 Radiotherapy & OncoImmunology Laboratory, Department of Radiation Oncology, Radboud Institute for Molecular Life Sciences (RIMLS), Radboud University Medical Center, 6525 GA Nijmegen, The Netherlands; Paul.Span@radboudumc.nl; 3 Department of Laboratory Medicine, Radboud University Medical Center, 6525 GA Nijmegen, The Netherlands; 4 Cancer Genomic Centre, University Medical Center Utrecht, 3584 CG Utrecht, The Netherlands; 5 David H. Koch Center for Applied Research of Genitourinary Cancers, The University of Texas MD Anderson Cancer Center, Houston, TX 77230, USA

**Keywords:** adherens junctions, p120catenin, breast cancer

## Abstract

Tumor metastasis is the endpoint of tumor progression and depends on the ability of tumor cells to locally invade tissue, transit through the bloodstream and ultimately to colonize secondary organs at distant sites. P120 catenin (p120) has been implicated as an important regulator of metastatic dissemination because of its roles in cell–cell junctional stability, cytoskeletal dynamics, growth and survival. However, conflicting roles for p120 in different tumor models and steps of metastasis have been reported, and the understanding of p120 functions is confounded by the differential expression of p120 isoforms, which differ in N-terminal length, tissue localization and, likely, function. Here, we used in silico exon expression analyses, in vitro invasion assays and both RT-PCR and immunofluorescence of human tumors. We show that alternative exon usage favors expression of short isoform p120-3 in 1098 breast tumors and correlates with poor prognosis. P120-3 is upregulated at the invasive front of breast cancer cells migrating as collective groups in vitro. Furthermore, we demonstrate in histological sections of 54 human breast cancer patients that p120-3 expression is maintained throughout the metastatic cascade, whereas p120-1 is differentially expressed and diminished during invasion and in metastases. These data suggest specific regulation and functions of p120-3 in breast cancer invasion and metastasis.

## 1. Introduction

P120 catenin (hereafter p120), which regulates adherens junctions by preventing the endocytosis of cadherins [1], has been identified as a regulator of tumorigenesis and tumor cell migration. Expression deregulation or mislocalization of p120 is observed in many different cancers [2,3,4,5]. Loss of p120 leads to loss of E-cadherin [1,6,7], which is thought to promote dissemination of cancer cells [8,9] and correlates with poor prognosis [10]. Conditional deletion of p120 promotes tumor formation in mouse models in several studies [11,12,13,14,15], indicating a tumor suppressor function of p120. However, positive contributions of p120 to cancer progression have also been reported [16,17]. These opposing observations may be due to the integrated effects of the many processes that are controlled by p120, including anchorage-independent growth, Wnt signaling, regulation of Rho GTPases, and promotion of migration and invasion [2,17,18,19]. P120 may promote cancer progression by facilitating E-cadherin-dependent collective migration of tumor cells [20,21,22]. Collective invasion of breast cancer cells into the mammary stroma is the prevalent invasion mode in breast cancer and correlates positively with metastatic outcome and worsened prognosis [23,24,25]. Thus, a functional p120/E-cadherin axis may contribute to tumorigenesis and support collective cancer progression and metastasis. 

One of the mechanisms by which p120-dependent pro- and antitumorigenic functions may be balanced is through alternative splicing. Alternative splicing is widespread in tumors and leads to expression of cancer type-specific protein isoforms [26]. In p120, alternative splicing leads to N-terminal variants, p120-1 through p120-4, according to usage of alternative start codons [27]. The most common isoforms, p120-1 and p120-3, are differentially expressed depending on tissue location and differentiation status [27,28,29]. P120 isoforms differ in their ability to regulate RhoGTPase [30] and transcription factor signaling [31,32,33], and differentially impact cell migration and proliferation [30,34]. Alternative splicing of p120 is regulated by several pathways which are also involved in epithelial differentiation and EMT and cancer progression. EMT transcription factors including Slug and Snail [6,35] promote expression of p120-1, whereas the action of ESRP-1 and -2, which directly mediate alternative splicing of p120-3 [36] as part of a general epithelial-promoting splicing program, is abrogated during EMT [37,38,39]. Thus, alternatively spliced p120 isoforms may be involved in seemingly conflicting p120 functions during cancer progression.

We here investigated whether p120 isoform switching occurs during ductal breast cancer progression. Furthermore, as p120 potentially fulfills different functions during different stages of tumor progression [18], we compared p120 isoform expression in breast cancer cells in different metastatic stages, i.e., in-situ carcinoma, locally invading cells, intravasated cells and lymph node metastases. We report increased p120-3/p120-1-encoding mRNA ratios in breast cancer, correlating with tumor grade, size and patient survival. Using novel isoform-specific antibodies [29] we demonstrate upregulation of p120-3 in invading tumor cells fronts in vitro. Analyses of human tumor sections demonstrate that p120-3 is consistently highly expressed in invasive tumor regions and throughout the metastatic cascade, whereas p120-1 expression depended on the metastatic stage and was mostly observed in non-invasive growth and intravascular transit. Our data show that p120 isoforms are expressed differentially during tumor progression, suggesting isoform-specific functions.

## 2. Materials and Methods

### 2.1. Cell Culture

The mouse mammary tumor cell lines 4T1 and 4TO7 were developed by Fred Miller, Michigan Cancer Foundation, Detroit, and kindly provided by Keltouma Driouch, Institute Curie, Paris. Cells were cultured at 37 °C, 5% CO_2_ in RPMI 1640 (Gibco, Gaithersburg, MD, USA) with 10% FCS, 100 U/mL penicillin/streptomycin and 1 mM sodium pyruvate. NMuMG cells were cultured at 37 °C and 10% CO_2_ in DMEM with 10% FCS, 10,000 U/mL penicillin/streptomycin, 2 mM L-glutamine and 10 µg/mL insulin. The identity of 4T1 cells was confirmed by short tandem repeat DNA profiling (IDEXX BioResearch, Hoofddorp, the Netherlands). Cells were regularly tested for mycoplasma infection using the MycoAlert^TM^ Mycoplasma Detection Kit (Lonza, Basel, Switzerland). 

### 2.2. Antibodies and Dyes

The following antibodies were used for Western blot of mouse cell lysates: mouse anti-pan-p120 (BD610134, 0.25 µg/mL), mouse anti-E-cadherin (BD610182, 0.5 µg/mL), rabbit polyclonal anti-α-catenin (Sigma-Aldrich, St. Louis, MO, USA); C2081, diluted 1:1000), rabbit anti-β-catenin (Santa Cruz sc-7199, 0.2 µg/mL), rabbit anti-β-actin (Cytoskeleton Inc, Denver, CO, USA, AAN-01, 0.5 µg/mL), rabbit anti-GAPDH (Sigma-Aldrich G9545, 0.2 µg/mL), and mouse monoclonal anti-β-tubulin (E7, Developmental Studies Hybridoma Bank, University of Iowa, IA, USA). Secondary antibodies used for Western blot were Alexa Fluor 680-conjugated or Alexa Fluor IRDye 800-conjugated goat anti-mouse and goat anti-rabbit IgG (Invitrogen/Thermofisher, Merelbeke, Belgium).

For immunofluorescence of mouse cells and human tissues, the following antibodies were used: mouse monoclonal 6H11 antibody specifically recognizing the longer p120 isoforms p120-1 and p120-2 (Santa Cruz, Santa Cruz, CA, USA, sc-23873, 0.4 µg/mL for cultured cells and 4 µg/mL for tissue sections), rabbit anti-p120-3 raised against the N-terminus of p120 [29], ImmunoGlobe, Himmelstadt, Germany, (5 µg/mL for all conditions), mouse anti-, p120 (BD610134, 0.5 µg/mL for cultured cells), rat anti-E-cadherin (DECMA-1, Sigma-Aldrich U3254, diluted 1:200 for cultured cells), mouse anti-E-cadherin (BD610182, 2.5 µg/mL for tissue sections), chicken anti-vimentin (Ab24525, diluted 1:400 for tissue sections) and wide spectrum Cytokeratin (PCK, Abcam, Cambridge, UK, yAb9377, diluted 1:200 for tissue sections). Secondary antibodies were all Alexa Fluor™-conjugated and obtained from Invitrogen/Thermofisher, Merelbeke, Belgium and comprised 546Alexa Fluor- or 647 Alexa Fluor goat anti-mouse IgG (H + L), Alexa Fluor 546- or Alexa Fluor647 goat anti-rabbit IgG (H + L), Alexa Fluor 488 goat anti-chicken IgY (H + L) and Alexa Fluor 555 donkey anti-rabbit IgG (H + L) conjugates. Nuclei were stained with 4′,6-diamidine-2′-phenylindole dihydrochloride (DAPI, Roche 10236276001, 5 µg/mL) and F-actin by Alexa Fluor 488/568-conjugated Phalloidin (Invitrogen/Thermofisher; 1:200). 

### 2.3. Subcellular Protein Fractionation

Cells, grown to confluence in 6-cm culture dishes, were washed twice with PBS, scraped in PBS on ice, pelleted (5 min, 100 g, 4 °C), and resuspended in ice-cold buffer containing 3 mM Imidazole and 300 mM sucrose at pH 7.5, supplemented with protease and phosphatase inhibitors (5 µg/mL pepstatin, 10 µg/mL chymostatin, 3 µg/mL leupeptin, 10 µg/mL antipain, 0.5 mM benzamidine, 0.2 mM PMSF, 0.1 kU/mL aprotinin, 1 mM Na_3_VO_4_, 1 mM NaF). The cell membranes and cytosol were separated from the nuclei by passing the cells through a 25 gauge needle until completion as confirmed by visual inspection. The nuclei were subsequently sedimented (5 min, 14 × 10^3^ g, 4 °C) and the supernatant was collected. Membrane and cytosol fractions were separated by centrifugation (1 h, 105 × 10^3^ g, 4 °C; Sorvall WX80 A98 centrifuge with an SW60 Ti rotor). Samples were diluted with concentrated Laemmli buffer (100 mM Tris-HCl, 4% SDS, 20% glycerol, 200 mM DTT, bromophenol blue), boiled (5 min, 95 °C) and analyzed by Western blot. Approximately 10% of the sample was loaded as total lysate.

### 2.4. Cytoskeleton Extraction

Cells grown to confluence in a 12-well-plate culture were placed on ice, washed twice with ice-cold PBS++ (PBS with 0.5 mM Mg^2+^ and 1 mM Ca^2+^), and incubated 20 min on ice with 250 µL extraction buffer (50 mM NaCl, 300 mM sucrose, 10 mM Pipes, pH 6.8, 3 mM MgCl_2_, 0.5% (*v/v*) Triton X-100 supplemented with protease and phosphatase inhibitors as above). Next, the extracted cytosol was removed and diluted with concentrated Laemmli buffer as described above. The remaining cytoskeletal fraction was washed twice with ice-cold PBS++, and subsequently scraped in Laemmli buffer and analyzed by Western Blot.

### 2.5. Co-Immunoprecipitation with p120 Isoform-Specific Antibodies

Cells grown to confluence in 10-cm culture dishes were washed twice with ice-cold PBS and subsequently scraped on ice with 1 mL IP lysis buffer (125 mM NaCl, 20 mM Hepes pH 7.4, 1% Nonidet P40 substitute, supplemented with protease and phosphatase inhibitors as above). Cellular debris was removed by centrifugation (15 min, 15 × 10^3^ *g*, 4 °C) and pre-cleared with CL-4B beads (GE Healthcare, Piscataway, NJ, USA) for 30 min at 4 °C. 5% of the cleared lysate was taken as a total lysate control. The remaining lysates were incubated sequentially with 1 µg of antibody (1 h, 4 °C) and Sepharose-Protein G (16 h, 4 °C while rotating). The beads were washed five times with IP lysis buffer by centrifugation. Proteins were eluted from the beads by boiling in Laemmli buffer (5 min, 95 °C) and analyzed by Western blotting. To compare co-immunoprecipitation of the target proteins with p120-1 and p120-3, two-tailed Student’s *t*-tests were performed. The variances were determined not to be significantly different using an *F*-test.

### 2.6. Western Blot Analysis

Proteins were separated by size by means of SDS-PAGE, and subsequently transferred to PVDF membrane. Unspecific binding sites on the blots were blocked with PBST (PBS with 0.2% Tween-20) with 5% BSA for 1 h and the blots were incubated with primary antibodies in 5% BSA in 0.2% PBST overnight at 4 °C. Subsequently, the blots were washed six times with 0.2% PBST and incubated with Alexa Fluor-conjugated secondary antibodies (Invitrogen/Thermofischer, Merelbeke, Belgium) for 1 h. The blots were washed again six times with 0.2% PBST and once in PBS. Fluorescent scans of the blots were made using the Odyssey CLx imaging system (Li-Cor, Lincoln, NE, USA) and analyzed with the Image Studio Lite software Version 4.0. Uncropped versions of all the blots are shown in Figure S8 with integrated densities of the bands indicated in the blots.

### 2.7. Immunofluorescence of Cultured Cells

Cells on coverslips were pre-incubated with 10% normal goat serum in PBS with 0.3% Triton X-100 (1 h) to block unspecific binding sites, washed with PBS, and incubated with primary antibody for 2 h. After washing with PBS, samples were incubated with fluorophore-conjugated secondary antibody and probes for 1 h in 5% BSA and 0.3% Triton X-100 in PBS, and then washed with PBS. The coverslips were mounted on object slides with FluorSave (Sigma-Aldrich). Cells were imaged with the Olympus FluoView 1000 confocal microscope, using a UPLSAPO 60× oil objective.

### 2.8. Gap Closure Assay

4T1 cells were grown to confluence inside silicone inserts (Ibidi, Gräfelfing, Germany) mounted on sterile coverslips. To initiate migration, the inserts were removed. After culture for 12 h samples were fixed (4% paraformaldehyde in 0.1 M phosphate buffer, pH 7.4; 15 min, 37 °C), washed three times with PBS, and stained for p120 isoforms, nuclei and F-actin as described above. Quantification of p120 expression as a function of distance from the leading front in the gap closure assays was performed using a semi-automatic custom written analysis script for Fiji/ImageJ (University of Wisconsin, Madison, WI, USA) (version used was Fiji 1.49m) [40]. Identification of the leading front was performed by first thresholding the actin image using the “MinError” threshold. Subsequently, small areas within the cell layer that were initially not thresholded were removed by a predefined number of erosion steps followed by as many dilation steps resulting in a well-defined binary mask for the area that was occupied by the cells. Next, an outline of this mask was created by the binary “outline” function and for the last step, the part of the outline at the image boundary was removed to only leave a line that defines the invasive front. Next, this line was moved away from the leading front into the cell layer and the average fluorescence intensity was calculated for the p120-1 and p120-3 channels at predefined steps (0.2 µm) over a predefined distance (180 µm) (Figure S4C). Images of confluent cell layers located at least 1000 µm away from the leading edge were taken as control. Fluorescent profiles of the distribution of p120-isoforms and actin were analyzed with Fiji/ImageJ 1.49m [40] and analyzed statistically using a Kruskal–Wallis test with a Dunn’s correction for multiple comparison.

### 2.9. Spheroid Invasion in 3D Culture 

P120 isoform distribution in invading tumor spheroids was done essentially as described [41]. Briefly, multicellular spheroids of 1000 4T1 cells were assembled for 24 h using the hanging-drop assay (20% Methylcellulose (Sigma)). After washing with PBS, spheroids were embedded in 4 mg/mL non-pepsinized rat-tail type I collagen (BD Biosciences, Franklin Lakes, NJ, USA). Spheroids were allowed to invade for 72 h and were then fixed for immunofluorescent staining for 15 min, using 4% PFA, at 37 °C. After washing, collagen gels were incubated with primary and secondary antibodies in PBS/BSA (0.1%)/Triton X-100 (0.3%) for 2–3 h at room temperature and imaged as whole-mount 3D samples. Confocal fluorescence and reflectance microscopy were performed by sequential single-channel confocal using an Olympus FV100 with a 40× objective.

### 2.10. In Silico Splicing Analysis of Human Breast Cancer RNA-seq Data

To plot the read distribution over exons of the p120 catenin gene (*CTNND1*) in human non-diseased and breast cancer tissue, the TCGA SpliceSeq was used (http://projects.insilico.us.com/TCGASpliceSeq, In Silico Solutions). Raw RNAseq data of 1094 breast cancer patients and 113 matched samples of adjacent normal tissue from TCGA-BRCA databases (https://portal.gdc.cancer.gov/projects/TCGA-BRCA) was retrieved and aligned with known exons. The number of exon observations per case was normalized over exon length in kb and million base pairs of total reads, and subsequently averaged per category, i.e., normal or tumor, and normalized over exon counts for the entire gene, to correct for gene expression differences and compare exon expression between breast cancer tissue and normal tissue controls.

### 2.11. P120 Isoform Expression in Human Breast Cancer Samples

Primary breast tumor and lymph node metastasis samples from human breast cancer patients that had not received adjuvant therapy were obtained, with informed consent, from the Radboud Breast Cancer Biobank, approval 2013/576 IRB (Institutional Review Board) Radboud University Medical Center. P120 isoform RNA expression was analyzed in primary tumor tissue. RNA was isolated using the Norgen’s total RNA purification kit (Norgen Biotek Corp, Thorold, ON, Canada) and stored at –80 °C. RNA quality was confirmed by A260/A280 and 28S/18S. The Reverse Transcription System (Promega, Madison, WI, USA) was used for cDNA synthesis. RT-PCR was performed using the equivalent of 50 ng RNA with 500 nM forward and reverse primers, in SYBR Green master mix (Biorad, Hercules, CA, USA) using a PTC-200 (MJ Research, Reno, NV, USA). The following variant-encompassing primers were used: forward 5’-TGCCCTGCTGGATTTGTCTT-3’, reverse 5’-CGAGTGGTCCCATCATCTG-3’ [27]. RT-PCR was performed using a PTC-200 (MJ Research). Samples were denatured (5 min, 94 °C), followed by 30 cycles of 40 s at 94 °C, 59 °C and 72 °C and a final extension step (5 min, 72 °C). PCR products were separated using a 1.2% agarose gel, along with a 100 bp ladder (New England BioLabs, Ipswich, MA, USA). PCR products were assigned to p120 isoforms by amplicon size and analyzed by densitometry (Figure S1B). A non-parametric Kruskal–Wallis test was used with a post-hoc Dunn’s test for multiple comparison of tumor size correlation with p120 isoform ratio, and a Spearman correlation was used for grade, as grade is an ordinal quantity. To compare survival, a Mantel–Cox test was used.

### 2.12. H&E and Immunofluorescence of Histological Sections

FFPE sections (4 µm) from human tissue from patients described above were deparaffinized and dehydrated in ethanol dilution series. For histological analysis, H&E staining was performed by incubating 20 min with hematoxylin and 5 min with eosin. For epitope retrieval and immunofluorescence staining, tissue sections were incubated in Tris-EDTA buffer (10 mM Tris, pH 9.0, 1 mM EDTA; 15 min, 95 °C), cooled in the same buffer for 1 h at room temperature, and washed with PBS for 1 h on a shaker. Non-specific epitopes were blocked with 10% NGS, 1% BSA and 0.2% Triton X-100 in PBS, followed by incubation with primary antibody overnight at 4 °C and secondary antibody and DAPI in 1% BSA/0.05% PBST for 1 h at room temperature. Adjacent tissue sections were incubated with species-matched non-specific IgG antibody as negative control. The sections were washed three times 5 min with 0.05% PBST after each antibody incubation step. Coverslips were mounted onto the sections with Fluoromount-G (ThermoFischer). The sections were imaged using either a Pannoramic Flash 250III microscope (3D Histech. Budapest, Hungary), using a Plan-Apochromat 20× objective and a CIS VCC-FC60FR19CL camera for H&E-stained samples and a pco.edge 5.5 4MP camera to detect (immuno)fluorescence, or a DMI600B microscope (Leica, Wetzlar, Germany)fitted with an HCX PL S-APO 20.0 × 0.50 DRY objective or an HCX PL S-APO 40.0 × 0.75 DRY objective and a DFC360FX-377550509 camera.

### 2.13. Analysis of p120 Isoform Expression in Human Breast Cancer Sections

Images of human breast tumor tissue sections were analyzed with the CaseViewer 2.0 software (3DHistech, Budapest, Hungary). Different histological subregions, including histologically normal mammary gland epithelium, carcinoma in situ, local invasion into the fibrous or adipose tissue, or intravascular cells were independently scored in a blinded manner by two researchers for high, intermediate or absent fluorescence intensity of p120-1, p120-3 and E-cadherin (Figure S6). To account for the non-Gaussian distribution of data, a non-parametric Kruskal–Wallis test was used with a post-hoc Dunn’s test for multiple comparison. 

## 3. Results

### 3.1. High p120-3/p120-1 Isoform Expression Ratio Predicts Poor Prognosis

**Figure 1:**
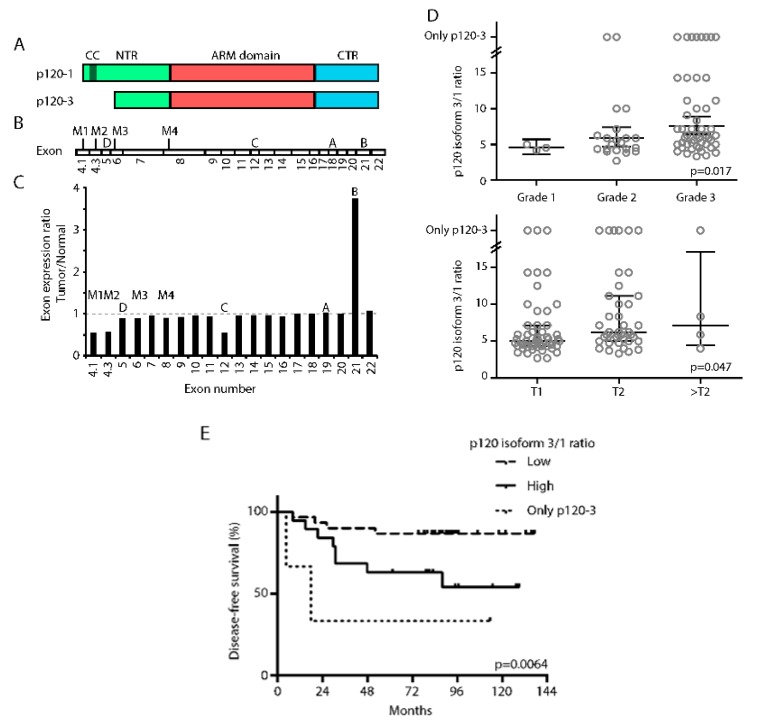
P120 isoform expression is changed in invasive breast cancer and correlates with poor prognosis. (**A**) Schematic overview of p120-1 and p120-3 protein structure. CC, coiled coil domain; NTR, N-terminal region; ARM, Armadillo repeat domain; CTR, C-terminal region. (**B**) Schematic overview of the exons in the *CTNND1* gene coding for p120 protein. The alternative entry points are indicated by M1, M2, M3 and M4, and the corresponding encoding alternative exons are indicated with the numbers below. The alternative exons A, B, C and D are indicated with their respective letters. (**C**) *CTNND1* exon expression ratios of tumor/normal mRNA from the TCGA-BRCA database. Alternative entry points and exons are indicated above the bars. (**D**) Correlation between p120-3/p120-1 isoform encoding ratios in human breast cancer tissues and pathological features. Significant positive correlations were found between p120-3/p120-1 isoform ratio and tumor grade (Spearman correlation, Rs = 0.217, *p* = 0.017) and size (Kruskal–Wallis, *p* = 0.047). (**E**) Significant differences in survival depending on p120-3/-120-1 isoform ratio of patients with T1 breast tumors (Mantel–Cox test, *p* = 0.0064).

P120 isoform mRNA expression depends on alternative splicing of *CTNND1* mRNA [27] (Figure 1A,B). There are four alternative entry points, designated M1–4, indicating the methionine residues encoded by the respective start codons. Inclusion of all entry points leads to translation from start codon M1 and subsequent formation of protein isoform p120-1, whereas exclusion of M1 and M2 by splicing in the 5’ region of the mRNA results in formation of isoform p120-3. Furthermore, *CTNND1* mRNA contains four alternative exons (termed A–D) which can be included or excluded independently. We first compared the exon expression levels between invasive breast cancer tissue and adjacent controls from the TCGA-BRCA cohort (*n* = 1098) with TCGA SpliceSeq (Table S1). Using this method, we could demonstrate a 3.2-fold higher inclusion of alternative exon B in cancerous compared to control tissue, which is consistent with previous reports [42,43] (Figure 1C and Figure S1A). In addition, our analyses revealed the novel observation that exons 4.1 and 4.3, containing the start codons M1 and M2, respectively, are twofold lower expressed in cancer tissue compared to tumor-free control tissue thus suggesting a higher ratio of p120-3/p120-1 encoding mRNA in cancer as compared to control tissue. These findings were surprising, as EMT transcription factors, whose presence is established in breast cancer [8], cause an isoform switch towards p120-1 in vitro [6,35]. We therefore subsequently examined the N-terminus of *CTNND1* transcripts in more detail in mRNA samples of a cohort of 96 breast cancer patients using variant-encompassing primers spanning the 5’ region containing the alternative entry points (Figure S1B). In this cohort, the p120 isoform 3/1 encoding ratio correlated positively with tumor grade and size (Figure 1D). Additionally, the p120-3/p120-1 encoding ratio negatively correlated with patient survival in T1 tumors (Figure 1E). No correlation was found between p120 isoforms and age, growth receptor levels or occurrence of lymph node metastasis. These data indicate that p120 isoform expression represents a prognostic factor in breast cancer.

### 3.2. P120 Isoforms Equally Associate with Adherens Junctions in Confluent Monolayers

**Figure 2:**
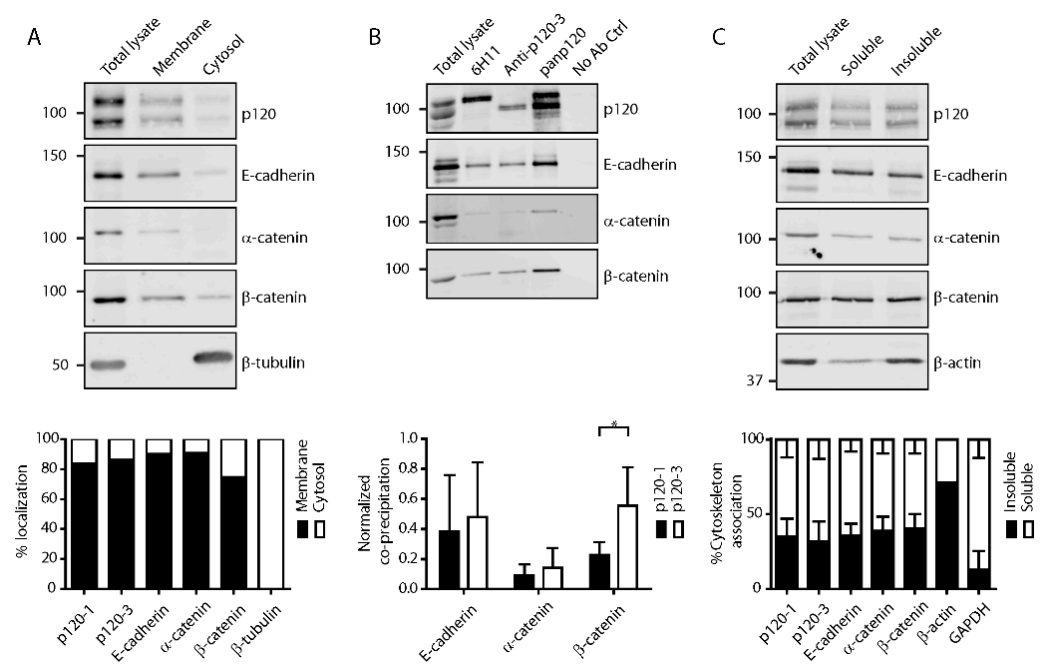
P120 isoform association with adherens junctions in 4T1 cells. (**A**) Membrane–cytosol fractionation. (**B**) Co-immunoprecipitation with isoform-specific antibodies for p120-1 (6H11 mAb) and p120-3 (anti-p120-3 pAb). IP antibodies are indicated above the blots (* *p* < 0.05). (**C**) Cytosol extraction. All membranes were incubated with the antibodies indicated adjacent to the blots. In the blots stained for p120, the upper band represents p120-1 and the lower band represents p120-3. Quantifications are shown in bar graphs below the blots.

P120 binding stabilizes classical cadherins at the cell membrane [1]. As previous studies have shown that both p120-1 and p120-3 bind to E-cadherin [44,45,46], we next addressed whether there are quantitative differences in p120 isoform association with cadherins. Fractionation of confluent 4T1 murine mammary cancer cells and normal murine mammary gland (NMuMG) cells into cytosol and membrane compartments showed that both p120 isoforms, as well as E-cadherin, α- and β-catenin are predominantly found in the membrane fraction (Figure 2A and Figure S2A). By contrast, in 4TO7 mesenchymal murine mammary tumor cells, which lack E-, N- or P-cadherin-based adherens junctions, both p120 isoforms predominantly localized in the cytosolic fraction (Figures S2B and S3). Thus, in mammary epithelial cells, membrane localization of both p120-1 and p120-3 depends on the presence of cadherins, consistent with previous reports [46]. To address directly whether p120-1 and p120-3 differentially interact with adherens junction proteins, we used p120 isoform-specific antibodies to precipitate p120 and associated proteins. Co-immunoprecipitation of E-cadherin and α-catenin did not differ between p120-1 and p120-3 although p120-3 precipitated significantly more β-catenin in 4T1 (Figure 2B and Figure S2C). Furthermore, reciprocal co-immunoprecipitation with an antibody against E-cadherin co-precipitated both isoforms at ratios equivalent to those expressed in total lysates (Figure S2D,E). These results confirm similar binding of p120-1 and p120-3 to E-cadherin and likely indirect association with α-catenin, which confirms the strong membrane localization of both p120 isoforms in confluent cell layers [29]. As adherens junction complexes require a link to the actin cytoskeleton for functional adhesion and mechanotransduction [47], we analyzed the cytoskeleton association of p120-1 and p120-3 and other adherens junction proteins, and detected no differential association in 4T1 cells (Figure 2C; ~35%) and in NMuMG cells (Figure S2F; ~55%). In 4TO7 cells lacking adherens junctions, p120 did not associate with the cytoskeleton (Figure S2G). These results indicate that in cells in a confluent monolayer p120-1 and p120-3 equally bind to adherens junctions and connect to the actin cytoskeleton.

### 3.3. Upregulation of p120-3 at the Invasive Front of Moving Multicellular Sheets

**Figure 3:**
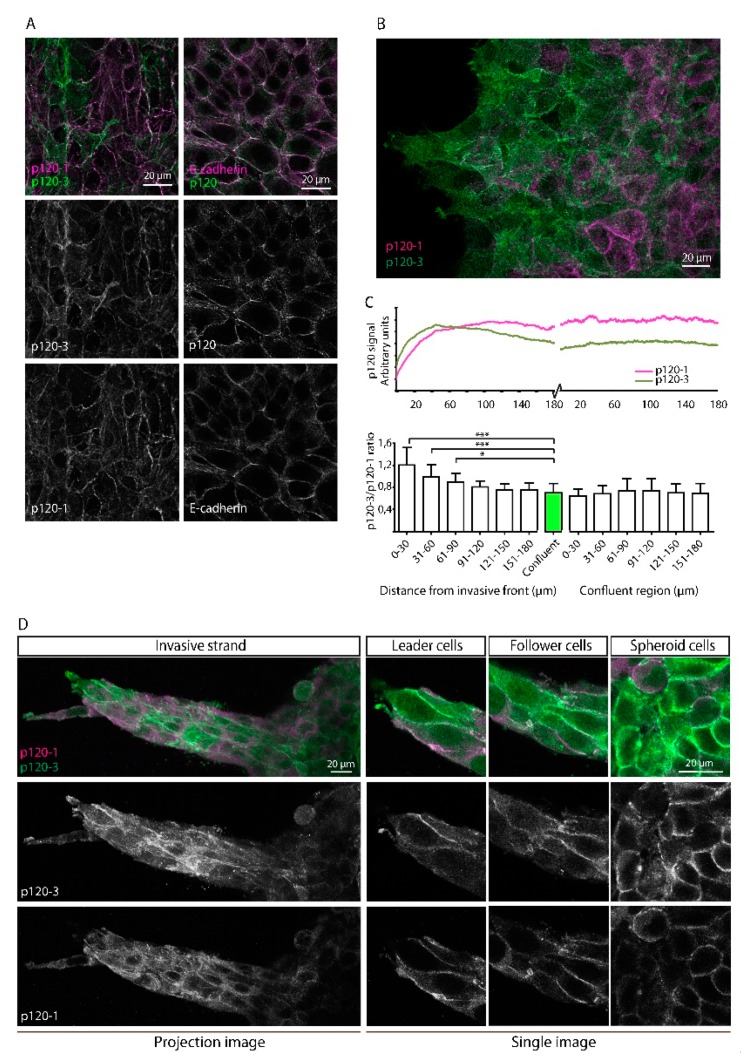
4T1 cells heterogeneously express p120 isoforms and preferentially express p120-at the invasive front. (**A**) Confluent 4T1 cells show heterogeneous expression of p120 isoforms upon immunostaining with p120-3 (anti-p120-3 pAb) and p120-1 (6H11 mAb) and homogeneous expression of total p120 (anti-p120 mAb) and E-cadherin (DECMA-1 mAb). (**B**) Immunofluorescent labeling of migrating 4T1 cells in a gap closure assay demonstrate preferential expression of p120-3 (anti-p120-3 pAb) as compared to p120-1 (6H11 mAb) at the leading front. (**C**) p120 isoform expression profiles, as quantified by distance from the invasive front. Lower panel shows the binned p120-3/p120-1 isoform ratios over intervals of 30 µm. The green bar in the lower panel represents the average of p120 isoform ratio in the confluent region. P120-3/p120-1 isoform ratios were compared using a Kruskal–Wallis test, and were significantly different between the first 90 µm from the invasive front and the average of the confluent region. * indicates *p* < 0.05, *** indicates *p* < 0.001. (**D**) p120 isoform expression in 4T1 cells migrating as invasive strands in 3D collagen. Panels show p120-1 (6H11 mAb) and p120-3 (anti-p120-3 pAb) in cells at the leading edge, follower cells and cells that had not migrated out of the spheroid.

We next aimed to determine p120 isoform localization in 4T1 cells. Both p120-1 and p120-3 exhibited strong membranous localization (Figure 3A). No nuclear signal was observed, which is in agreement with low steady-state levels of nuclear p120 reported previously [48]. The relative p120 isoform distributions differed strongly between individual 4T1 cells (Figure 3A), but not in NMuMG cells (Figure S4A). The intercellular heterogeneous expression of p120 isoforms was not seen for total p120 levels or E-cadherin (Figure 3A), indicating that adherens junction formation was not affected by heterogenous p120 isoform expression. To analyze p120 isoform expression distribution in migratory cells, 4T1 cells were allowed to migrate for 12 h in a gap closure assay. Immunolabelling of the migrated cells with p120 isoform-specific antibodies showed that p120-3 expression was elevated in cells at the leading front (Figure 3B). 

Quantification of p120 isoform expression (Figure 3B and Figure S4B,C) showed that p120-3 immunofluorescence was high in the first 60 µm from the leading edge, and gradually decreased to levels detected in non-migratory confluent cells. By contrast, p120-1 expression was low at the leading edge and increased until a plateau was reached 100 µm rearward from the leading edge, similar to staining intensity in a non-moving monolayer (Figure 3B,C). This differential expression of p120 isoform was reflected by a significantly higher p120-3/p120-1 isoform ratio at the first 90 mm of the invasive front compared to the trailing edge (Figure 3C). Similarly, in collective strands of 4T1 cells invading into a 3D collagen matrix, p120 isoform distribution was heterogeneous, and p120-3 was elevated near the leading edge (Figure 3D). These data indicate that local upregulation of p120-3 but not p120-1 is associated with the invasive front of invading breast cancer cells in 2D and 3D cell culture models.

### 3.4. P120-3 Is Expressed throughout the Metastatic Cascade, Whereas p120-1 Is Reduced in Invasive Regions

**Figure 4:**
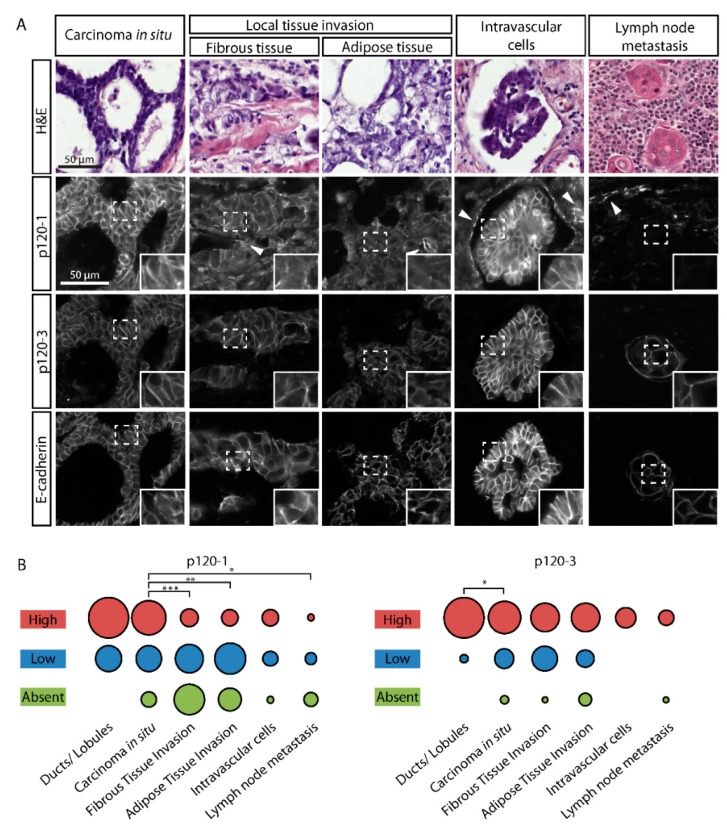
P120 isoform expression in different breast tumor subregions. (**A**) Immunofluorescent labeling of p120 isoforms in breast tumor subregions. Panels show p120-1 (6H11 mAb), p120-3 (anti-p120-3 pAb) and E-cadherin in ductal carcinoma in situ, local invasion into fibrous and adipose tissue, intravascular tumor cells and lymph node metastases. H&E of the subregions from an adjacent section is shown. Intravasated tumor cells or clusters were identified by the surrounding vessel wall from H&E and characteristic endothelial p120-1 expression (arrowheads). (**B**) Quantification of (**A**). * indicates *p* < 0.05, ** indicates *p* < 0.01, *** indicates *p* < 0.001.

To determine p120 isoform expression in invasive regions of human tumors, we examined histological sections of 54 human ductal breast cancer patients using isoform-specific antibodies. Non-invasive in-situ tumor cells in sections of human mammary tumors exhibited strong membranous signals of p120-1, p120-3 and E-cadherin, similar to histologically normal ducts and lobules adjacent to tumor tissue (Figure 4A and Figure S5). Predominantly collective local invasion zones located in the fibrous and adipose tissue showed high expression of membranous E-cadherin and p120-3, but not p120-1 (Figure 4A). We next scored p120 isoform expression based on immunofluorescence intensity (Figure S6) in tumor cells at subsequent stages of the metastatic cascade, i.e., intravasation into lymph or blood vessels and locoregional lymph node metastasis (Figure S7). High p120-3 expression and membrane localization, along with E-cadherin, were maintained in cell clusters present inside the vasculature and in lymph node metastases, whereas p120-1 reduction was statistically significant in local collective invasion zones and lymph nodes (Figure 4A,B). As a notable exception, p120-1 was strongly upregulated in intravascular cell clusters, which also co-expressed p120-3 and E-cadherin (Figure 4A). These data identify p120-3 as constitutive and p120-1 as adaptively expressed isoform during tumor progression.

## 4. Discussion 

By combining exon expression analysis, in vitro invasion assays and immunofluorescence of human tumors, we identify diminished p120-1 isoform expression during invasion and distant metastasis of ductal breast cancer progression, whereas p120-3 is upregulated at the invasive front of moving breast cancer groups in vitro and constitutively maintained at each step of metastasis. Differential p120 isoform expression identified here complements current concepts of p120 functions during metastatic cancer progression and suggests novel and previously underappreciated functions of p120-3 in metastatic cancer progression. 

By using a recently developed p120-3-specific antibody [29], we directly detected p120-3 expression in tumors in comparison with p120-1, an approach that was previously precluded by the lack of specific reagents. Previous studies aimed at analyzing p120 isoform expression in tumors applied indirect approaches using cell lines [49,50], histological patient sections [3,51] or patient protein and mRNA samples [30,52]. These studies reported both increased [30,51] and decreased p120 isoform ratios in cancer [3,52]. We report a differential isoform expression depending on tumor subregions, and this may explain the discrepancies between these studies. In addition, the reported high levels of nuclear p120-1 in breast cancer tissue [3] could not be confirmed using the direct labeling approach. Furthermore, it has been previously shown that breast cancer subtypes correlate with different p120 N-terminal variants [49,50] and alternative exon expression [42,43], demonstrating isoform-specific expression of p120 in different cancer types. Using our direct dual-isoform labeling approach, we now have been able to provide spatial information on p120-1 and p120-3 expression in a single cell in its native environment at different stages of metastasis.

We demonstrate that p120-3 is maintained during all steps of metastatic human breast cancer progression and p120-3 expression even increases at the invasive front in in vitro invasion assays. By contrast, p120-1 exhibits more variable expression in distinct phases of metastasis. These data suggest that beyond alternative splicing, additional regulatory mechanisms of expression of both isoforms exist, dependent on cell function and the microenvironmental context. Our observation of collectively migrating cells with membranous E-cadherin and p120-3 are consistent with the retention of E-cadherin-based junctions and prevalence of collective invasion in mouse models [23] and breast cancer [25] and other cancer patients [24]. Mechanistically, the molecular mechanisms controlling the regulation of p120 isoform expression remain poorly understood. Known regulators of p120 splicing include the epithelial splicing proteins ESRP-1 and -2, which induce a switch from p120-1 to p120-3 [36] and inhibit EMT [39]. The expression of both ESRP1 and ESRP2 is differentially regulated by cell density [53] and hypoxia [39] in breast cancer cells, suggesting local and microenvironmental control of p120 isoform expression. Other known regulators of p120 isoform expression include the EMT transcription factors Slug and Snail, which induce a switch from p120-3 to p120-1 [6,35]. Our data are thus not consistent with EMT-induced expression of p120-1, but rather indicate an alternative mechanism by which p120-3 is preferentially expressed. At the protein level, p120-1, but not p120-3, is subject to degradation by the Wnt destruction complex due to the destruction sequence that is located in the p120-1 N-terminus [54]. As we report here, β-catenin, which is an important intermediate in this pathway, preferentially associates with p120-3 in a Triton X100-soluble and thus non-cytoskeleton-associated fraction. Differential regulation of Wnt signaling at invasive fronts may contribute to site-specific p120-1 downregulation, and may also involve Kaiso, an inhibitor of Wnt signaling, which preferentially associates with p120-3 [31,33]. Future research will show how tumor cells and their microenvironment regulate p120 isoform expression during the different stages of metastasis. Such studies may also include alternative exon B, which was also upregulated in breast cancer cells, encodes a nuclear export sequence, and thus may affect the nuclear functions of p120 [55].

Considering the differential effects of p120-1 and p120-3 on invasion, proliferation and metastasis [21,30,34], the high expression of p120-3 and varying levels of p120-1 in different stages of metastasis may play important roles in controlling tumor progression, and this may be partially regulated by p120 isoform-specific downstream interaction partners. For instance, the p120-1 N-terminus mediates interaction with kinesin and RhoA [30,56], which may lead to differential regulation of membrane targeting and contractility. Additionally, p120-1 is required for cytokinesis and chromosomal stability in breast cancer cells [57], and in vitro proliferation [34], and thus retention of p120-1 may provide survival-promoting signals whereas loss of p120-1 might drive chromosomal instability and tumor development. The finding that p120-3 is a consistent parameter during the different stages of breast cancer suggests that common shared functions such as cadherin stabilization, previously assigned to any p120 isoform, may be exclusively mediated by p120-3. Even though both isoforms equally stabilize E-cadherin, adherens junctions based on p120-3, which lacks the N-terminal domain involved in RhoA regulation, may lead to different actomyosin contractility at the adherens junctions, and consequently to differences in their stability and mechanical properties [58]. Another interesting possibility is that p120-1 and p120-3 associate with different functional E-cadherin complexes, as a previous study demonstrated the existence of distinct apical and lateral complexes, where the apical complex associated with miRNA processing machinery, and the lateral complex with growth promotion through Src [59]. Furthermore, as we observe retention of p120-3 and loss of p120-1, a switch to p120-3 as dominant p120 isoform may be accompanied by a reduction in total p120 levels, which is in agreement with previous studies that show reduced total levels of p120 in cancer [2]. Thus, in addition to a p120 isoform switch, reduced total p120 levels may also lead to altered metastatic properties. Indeed, recent reports show that reduced levels, but not a total ablation of p120 in mouse models are sufficient for E-cadherin-mediated adhesion, collective migration and metastatic colonization [60,61]. Intermediate levels of total p120, with specific retention of p120-3, may therefore represent an optimum for dynamic cell–cell junctions and cell kinetics facilitating efficient collective invasion and metastasis.

## 5. Conclusions

In summary, we demonstrate expression of p120-1 in specific stages of ductal breast cancer progression, whereas presence of p120-3 is maintained. Our findings may shed light on seemingly opposing reports regarding p120 tumor-suppressing and -promoting functions. As no differential interaction with E-cadherin was observed, it is likely that p120 isoforms fulfill E-cadherin-independent functions through differential binding partners. Future research focusing on isoform-specific downstream effects of p120 isoforms may identify therapeutically targetable pathways. As discussed above, several of these have been already identified for p120-1, and dysregulation of Rho-dependent signaling as a result of the loss of this isoform may be an avenue of targeted treatment. However, specific p120-3-dependent pathways are still largely unclear and the relevance for its maintenance during progression and its potential use as therapeutic target, in particular in the context of loss of p120-1, awaits further investigation.

## Supplementary Materials

The following are available online at https://www.mdpi.com/2072-6694/11/10/1459/s1, Table S1: Clinicopathological features of TCGA cohort, Figure S1: CTNND1 exon and isoform expression in breast tumors, Figure S2: p120 isoform association with other adherens components in NMuMG and 4TO7 cells, Figure S3: 4TO7 cells lack expression most of adherens junction components, Figure S4: p120 isoform expression in NMuMG and 4T1 cells, Figure S5: p120 isoform expression in normal mammary epithelium, Figure S6: Scoring examples of fluorescent intensity, Figure S7: Occurrence of metastatic events in breast cancer patient cohorts, Figure S8: Uncropped blots from Figure 2, Figure 3 and supplementary Figure S2, S3.
