# Supplementary material for: P120 Catenin Isoforms Differentially Associate with Breast Cancer Invasion and Metastasis
Source: Cancers (Basel). 2019 Sep 29;11(10):1459. doi: 10.3390/cancers11101459 (PMC6826419; doi:10.3390/cancers11101459)
Supplement: Supplementary file 1 [file cancers-11-01459-s001.pdf]

Supplementary Materials: P120 Catenin Isoforms Differentially Associate with Breast Cancer Invasion and Metastasis

Jan-Hendrik Venhuizen, Paul N. Span, Koen van den Dries, Sebastian Sommer, Peter Friedl and Mirjam M. Zegers

Table S1. Clinicopathological features of TCGA cohort (https://gdc.cancer.gov/clinical-data-elements).

**Number**	**1098**
Median age at diagnosis (years, range)	59.0 (26.6–90.0)
Vital status	
Alive	945
Dead	152
Median survival (years, range)	3.2 (0–20.4)
Not reported	1
Type	
Infiltrating ductal carcinoma	778
Lobular carcinoma	201
Mixed infiltrating ductal and other carcinoma	47
Mucinous adenocarcinoma	16
Metaplastic carcinoma	14
Other	42
Stage	
Stage i	90
Stage ia	86
Stage ib	7
Stage ii	6
Stage iia	358
Stage iib	257
Stage iii	2
Stage iiia	155
Stage iiib	27
Stage iiic	65
Stage iv	20
Stage x/ not reported	25

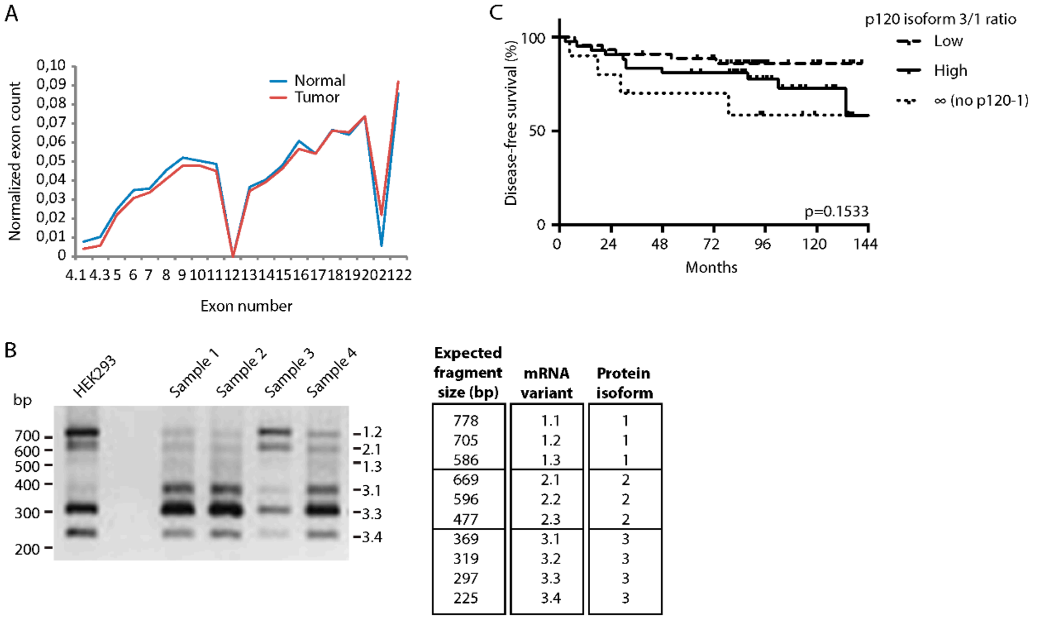

Expected fragment size (bp)	mRNA variant	Protein isoform
778	1.1	1
705	1.2	1
586	1.3	1
669	2.1	2
596	2.2	2
477	2.3	2
369	3.1	3
319	3.2	3
297	3.3	3
225	3.4	3

Figure S1. CTNND1 exon and isoform expression in breast tumors. (**A**) Normalized exon expression levels of breast tumor tissue and adjacent normal tissue from the TCGA database, analyzed with

TCGA SpliceSeq. (**B**) Analysis of 5′ transcript variants of p120 by RT-PCR in HEK293 cells and four human tumors using variant-encompassing primers [27]. Amplicons were assigned to a p120 isoform based on fragment size, as indicated in the table. Alternative splicing occurs in the 5′ UTR region of the mRNA, and thus mRNA variants 1.1, 1.2 and 1.3 all encode for isoform 1. (**C**) Correlation of p120 3/1 isoform-encoding mRNA expression in breast cancer patients with T1 and T2 tumors with cumulative survival (Mantel-Cox test, $p = 0.1533$).

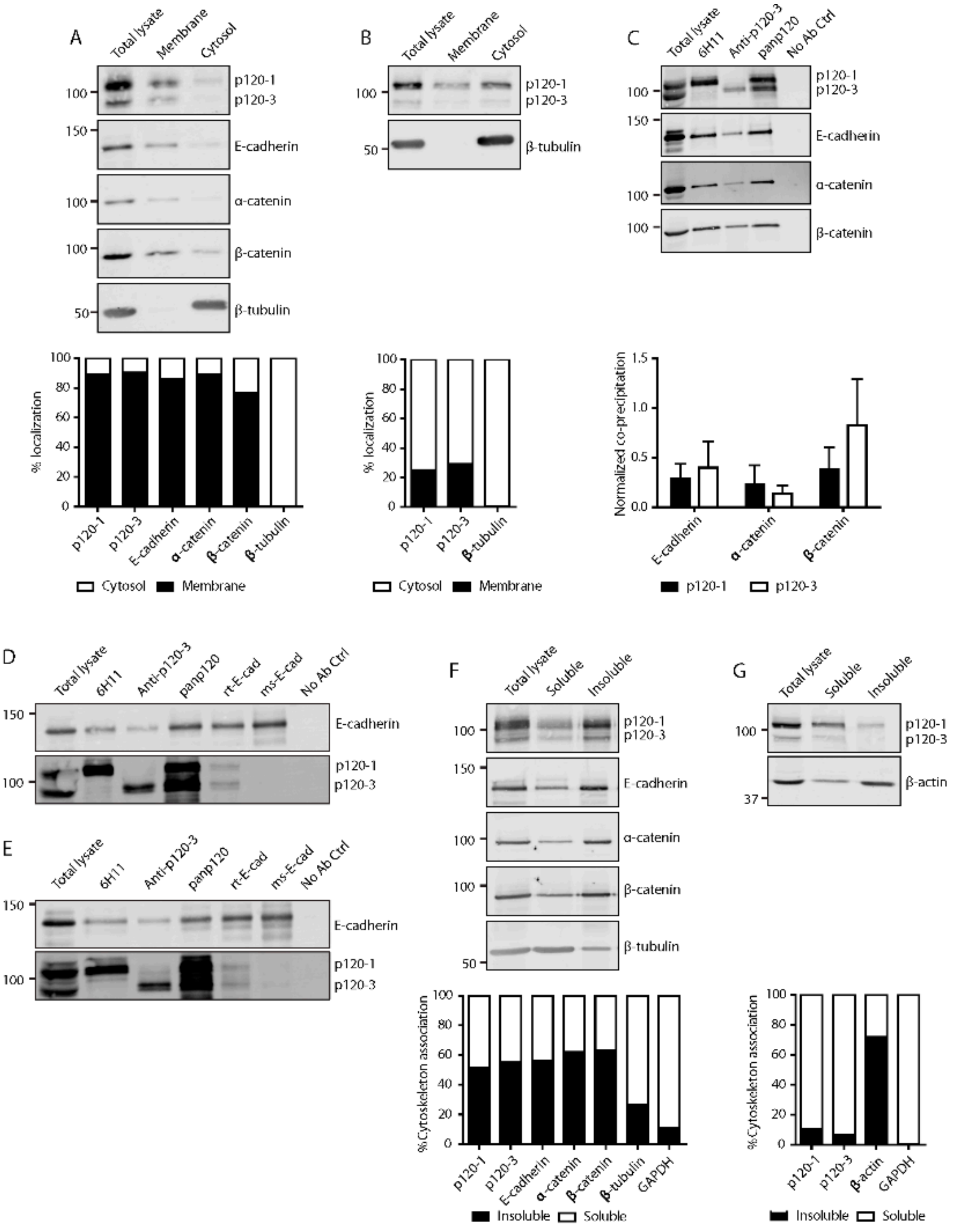

Figure S2. p120 isoform association with other adherens components in NMuMG and 4TO7 cells**. (A, B)** Membrane-cytosol fractionation in NMuMG and 4TO7 cells, resp. (C) Co-immunoprecipitation with isoform-specific antibodies for p120-1 (6H11 mAb) and p120-3 (anti-p120-3 pAb) in NMuMG cells. IP antibodies are indicated above the blots. (**D, E**) Co-immunoprecipitation with antibodies against p120 (6H11 mAb, anti-p120-3 pAb, anti-pan-p120 mAb) and E-cadherin (rat-anti-E-cadherin pAb, mouse-anti-E-cadherin mAb) from 4T1 and NMuMG cells, resp. IP antibodies are indicated above the blots. (**F, G**) Cytosol extraction in NMuMG and 4TO7 cells, resp. All membranes were

incubated with the antibodies indicated adjacent to the blots. Quantifications are shown below the blots.

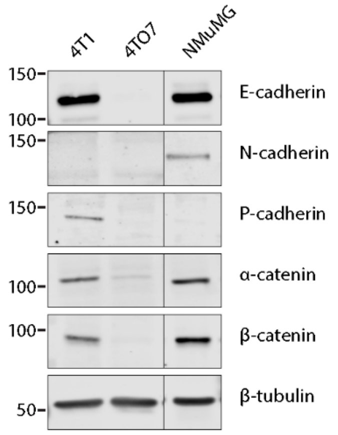

Figure S3. 4TO7 cells lack expression most of adherens junction components. The membranes were incubated with the antibodies indicated adjacent to the blots.

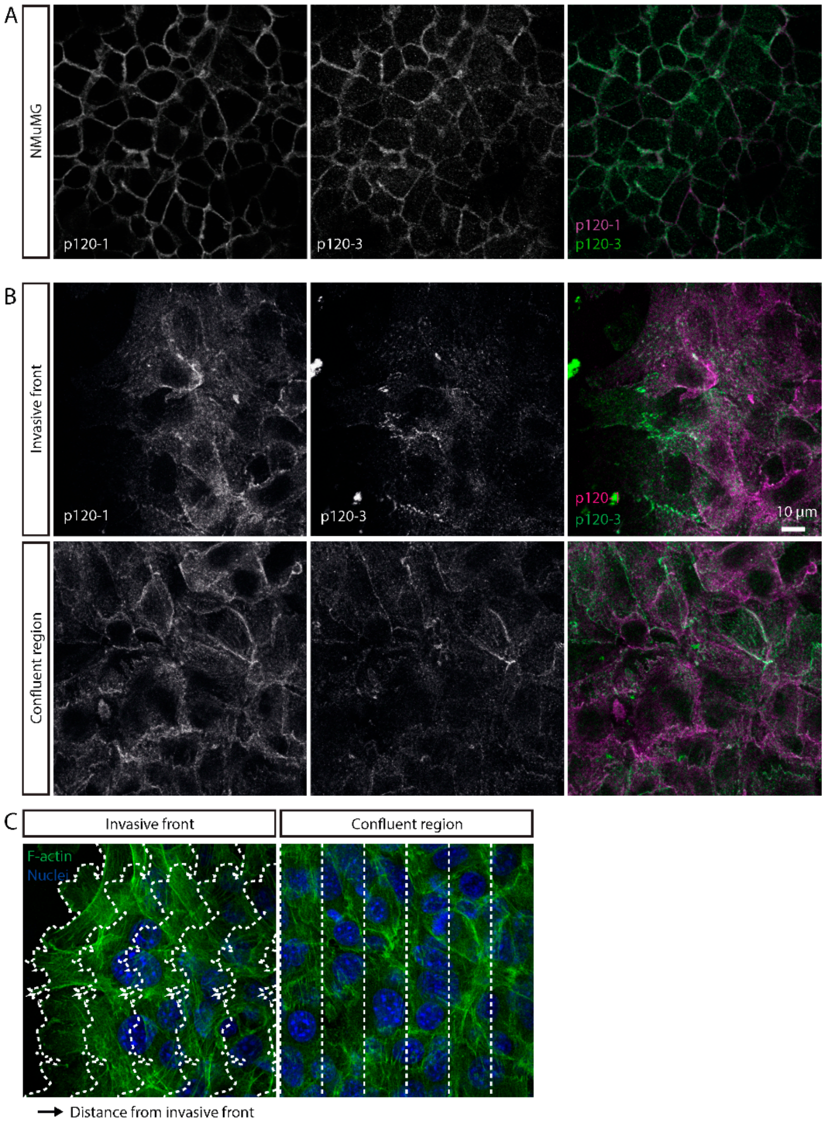

Figure S4. p120 isoform expression in NMuMG and 4T1 cells. (**A**) Expression of p120-1 and p120-3 in confluent NMuMG cells. (**B**) Expression of p120-1 and p120-3 in migrating 4T1 cells. Panels show 4T1 cells at the leading edge and confluent 4T1 cells in a region away from the leading edge. (**C**) Analysis of fluorescent intensity in migrating 4T1 cells. The leading edge was defined by F-actin signal, and p120 isoform expression was quantified over distance from the leading edge (dotted lines). In confluent 4T1 cells, isoform expression was quantified over distance from the edge of the frame.

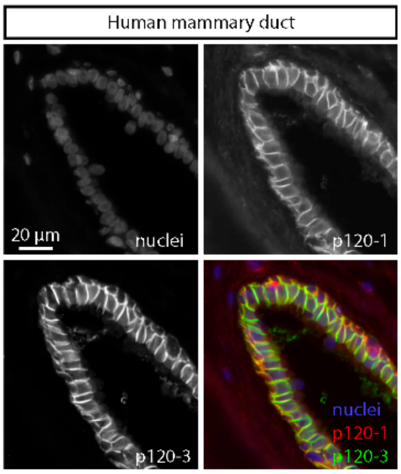

Figure S5. p120 isoform expression in normal mammary epithelium. Panels show p120-1 (6H11 mAb), p120-3 (anti-p120-3 pAb) and nuclei (DAPI) in human mammary ducts.

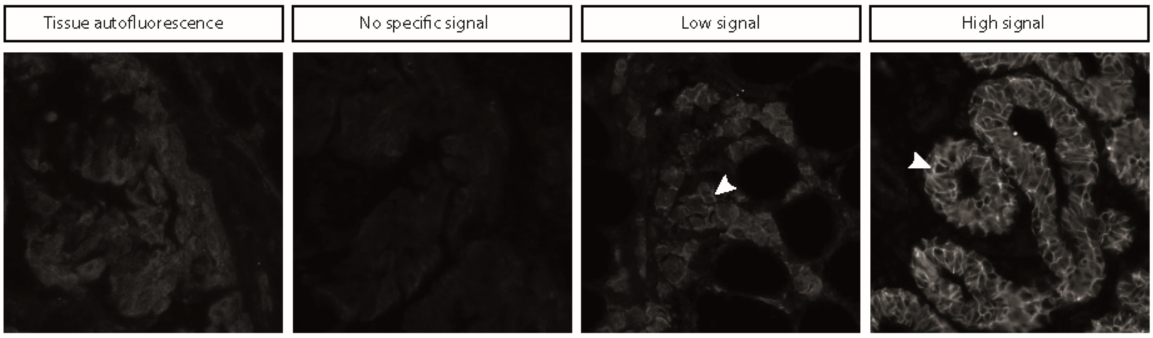

Figure S6. Scoring examples of fluorescent intensity. Fluorescent signals were compared to tissue autofluorescence and scored as 'absent', 'low' or 'high'. Arrowheads indicate fluorescent signal at the membrane.

A

	Carcinoma *in situ*	Local tissue invasion		Intravascular cells
		Fibrous tissue	Adipose tissue	
Stage reached as end point	6	7	29	12
Total occurences	44	47	38	12

Metastatic stage (no. of patients)

B

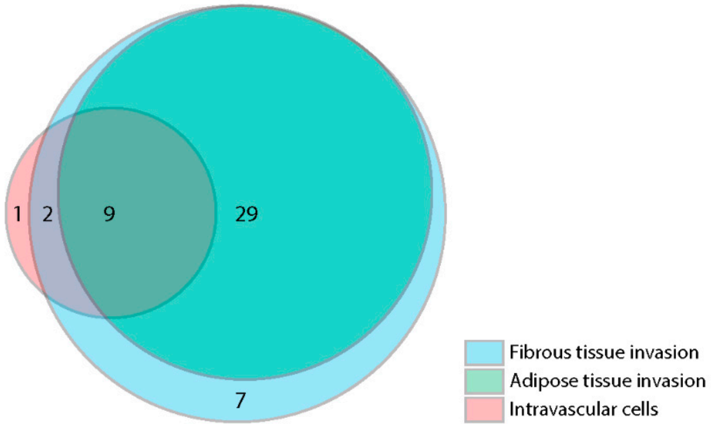

Venn diagram of metastatic stage (no. of patients)

Figure S7. Occurrence of metastatic events in breast cancer patient cohorts. (**A**) Most advanced metastatic stage observed in breast cancer patients, and total number of occurrences in the breast cancer patient cohort. (**B**) Venn diagram depicting the correlation between the occurrence of different metastatic events in breast cancer patients.

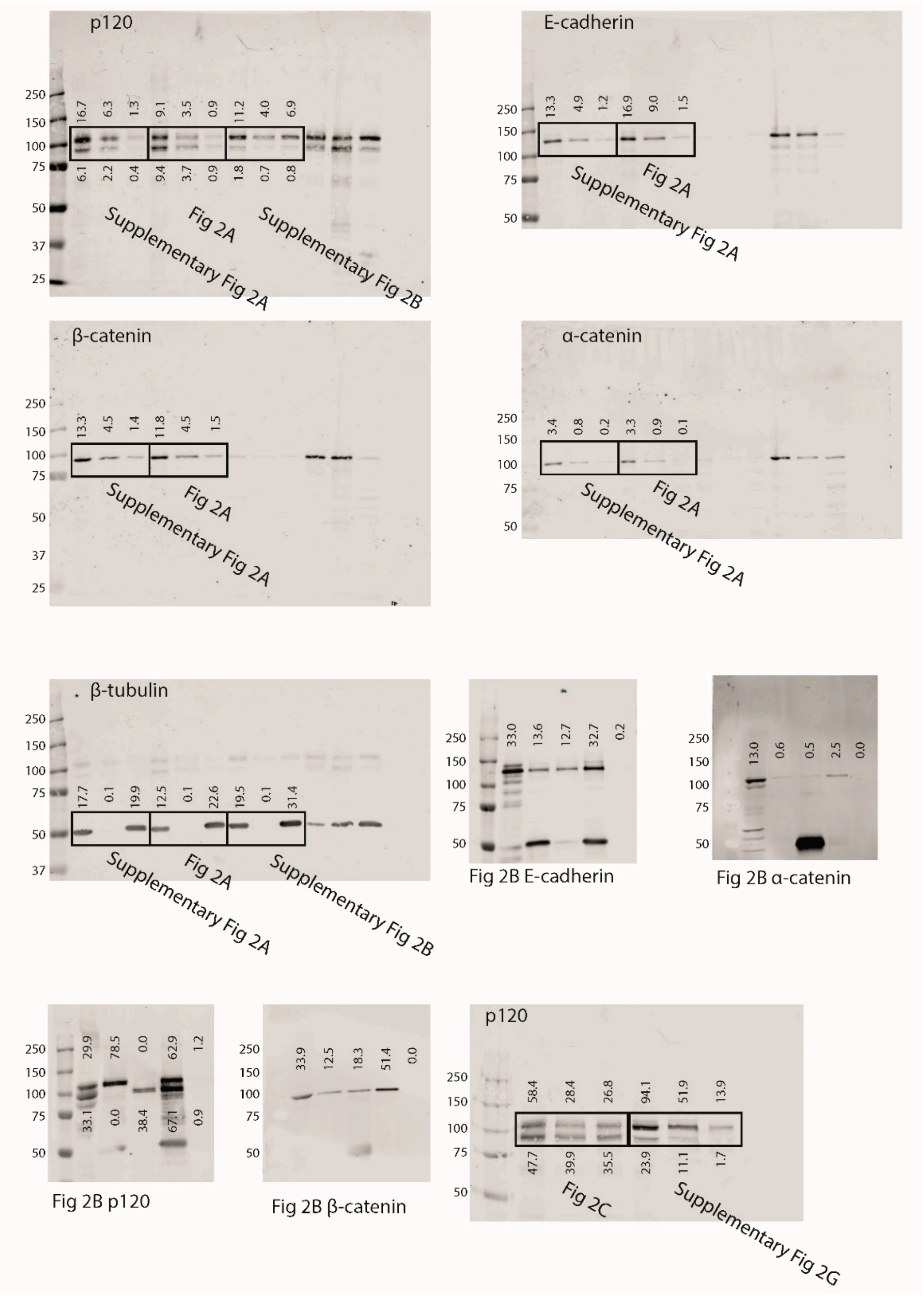
p120
250
150
100
75
50
37
25
16.7 6.3 1.3 9.1 3.5 0.9 11.2 4.0 6.9
6.1 2.2 0.4 9.4 3.7 0.9 1.8 0.7 0.8
Supplementary Fig 2A
Fig 2A
Supplementary Fig 2B
E-cadherin
13.3 4.9 1.2 16.9 9.0 1.5
Supplementary Fig 2A
Fig 2A
β-catenin
13.3 4.5 1.4 11.8 4.5 1.5
Supplementary Fig 2A
Fig 2A
α-catenin
3.4 0.8 0.2 3.3 0.9 0.1
Supplementary Fig 2A
Fig 2A
β-tubulin
17.7 0.1 19.9 12.5 0.1 22.6 19.5 0.1 31.4
Supplementary Fig 2A
Fig 2A
Supplementary Fig 2B
33.0 13.6 12.7 32.7 0.2
Fig 2B E-cadherin
13.0 0.6 0.5 2.5 0.0
Fig 2B α-catenin
29.9 78.5 0.0 62.9 1.2
33.1 0.0 38.4 67.1 0.9
Fig 2B p120
33.9 12.5 18.3 51.4 0.0
Fig 2B β-catenin
p120
58.4 28.4 26.8 94.1 51.9 13.9
47.7 39.9 35.5 23.9 11.1 1.7
Fig 2C
Supplementary Fig 2G

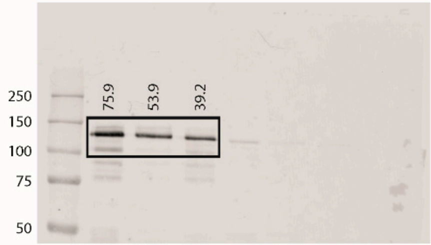

Fig 2C E-cadherin

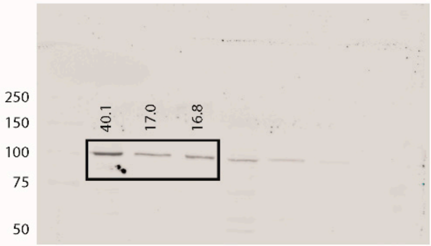

Fig 2C α-catenin

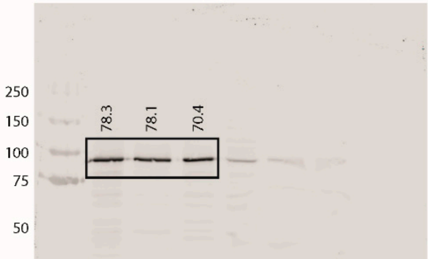

Fig 2C β-catenin

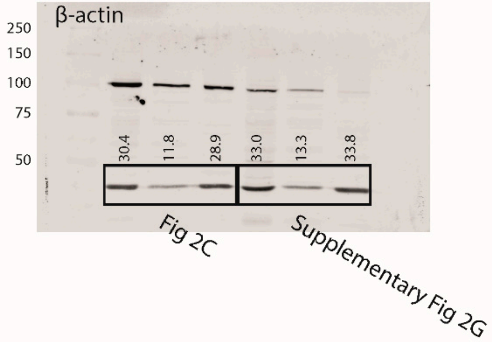

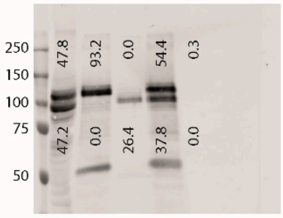

Supplementary Fig 2C p120

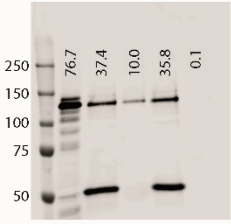

Supplementary Fig 2C E-cadherin

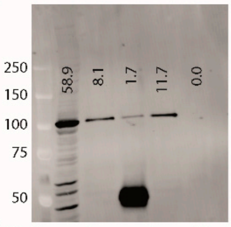

Supplementary Fig 2C α-catenin

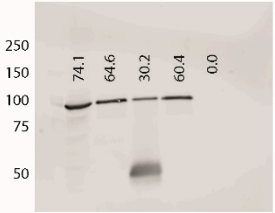

Supplementary Fig 2C β-catenin

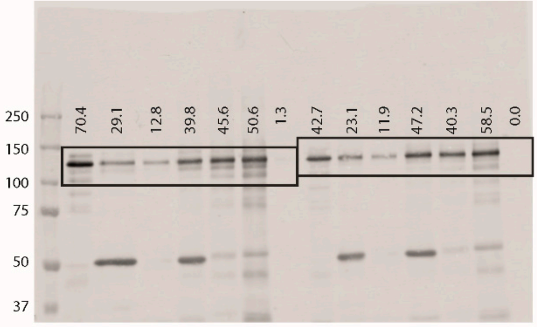

Supplementary Fig 2E E-cadherin

Supplementary Fig 2D E-cadherin

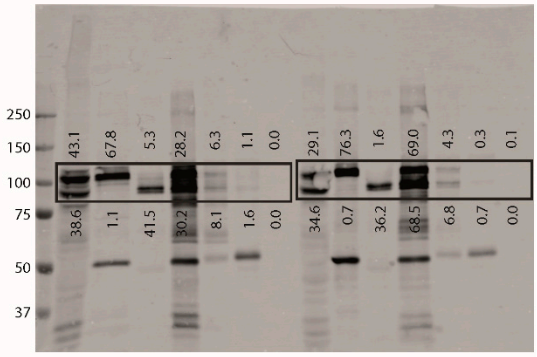

Supplementary Fig 2E p120

Supplementary Fig 2D p120

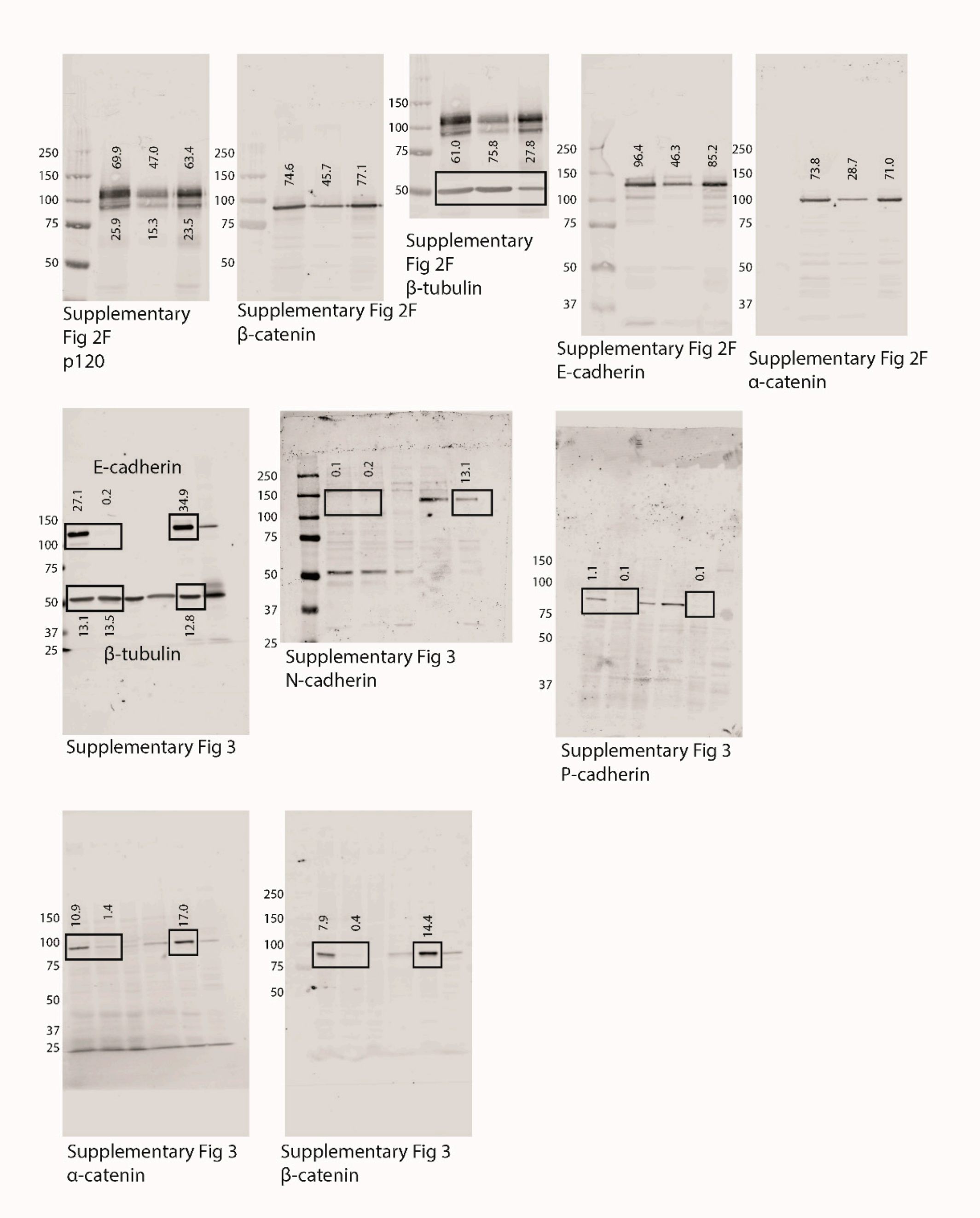

Figure S8. Uncropped blots from Figures 2, 3 and supplementary Figure 2, 3. Numbers next to the blot indicate molecular weight of the marker. Numbers on the blot indicate background-corrected integrated density of the individual bands.

© 2019 by the authors. Licensee MDPI, Basel, Switzerland. This article is an open access article distributed under the terms and conditions of the Creative Commons Attribution (CC BY) license (http://creativecommons.org/licenses/by/4.0/).
